# Using concept mapping to identify policy options and interventions towards people-centred health care services: a multi stakeholders perspective

**DOI:** 10.1186/s12939-018-0895-9

**Published:** 2018-12-04

**Authors:** Christine Cécile Leyns, Jan De Maeseneer, Sara Willems

**Affiliations:** 1 Asociación Interdisciplinaria de Atención Primaria de Salud – Bolivia, Calle Antezana N°686 esquina Salamanca, oficina 1D, Cochabamba, Bolivia; 2 Department of Public Health and Primary Care, Ghent University, Campus UZ Gent, 6K3, Corneel Heymanslaan 10, B-9000 Ghent, Belgium

**Keywords:** Policy, Professional competence, Stakeholder participation, Health equity, Health education

## Abstract

**Background:**

People-centred health care (PCC) services are identified by the WHO as important building blocks towards universal health coverage. In 2016 the WHO formulated a comprehensive framework on integrated PCC services based on an international expert consultation. Yet, expert opinions may fail to recognize the needs of all health system stakeholders. Therefore, a consultation method that includes the health workforce and laypersons, can be instrumental to elaborate this framework more in-depth. This research sought to identify participants’ perspectives on policy options and interventions to achieve people-centred health care services from a multi stakeholder perspective.

**Methods:**

Study participants, both laypersons and health professionals, were recruited in Belgium. A total of 53 participants engaged in one of the seven concept mapping workshops. In this workshop the concept mapping methodology developed by Trochim, a highly structured qualitative group method for brainstorming and idea sharing, was used to generate and structure participants´ perspectives on what is needed to achieve PCC services. The method was validated using the WHO framework.

**Results:**

The seven workshops together resulted in 452 different statements that were structured in a framework forming 35 clusters and four overarching domains. The four domains with their most prominent clusters were: (1) governance & policy with intersectoral health policies and affordable health for all; (2) health workforce with excellent communication skills, appreciation of health literacy challenges and respectful attitude based on cultural self-awareness; (3) integrated health services with a greater emphasis on prevention, health promotion and the availability of health education and (4) patient, person and community empowerment and participation with support for informal care, promotion of a healthy lifestyle and contextualised health education. Additionally, this study generated ideas that fitted into every single approach described in the WHO framework.

**Discussion and conclusion:**

This study shows that in order to achieve PCC a participative approach involving all stakeholders at all levels is needed. The concept mapping process is one of these approaches that brings together diverse stakeholders and foments their egalitarian and respectful participation. The framework that resulted from this study can inform future debate regarding planning, implementation and monitoring of PCC.

## Background

Since the WHO (World Health Organization) stated the importance of organising primary health care around people’s needs in its 2008 World Health Report [1], integrated people-centred health care (PCC) is considered the service model towards universal health coverage. This model surmounts the overall recognized patient centred care [2], towards the “person” [3], including prevention and health promotion and towards “people”, emphasizing the interconnectedness of persons, the importance of the community context and participation, the intersectoral nature of health [4] and the right to health (Universal Declaration of Human Rights, 1948). Although the people-centred health care concept was not widely used before, it largely resembles the comprehensive primary health care strategy expressed in the 1978 Declaration of Alma Ata [5].

To date, “People-centred health care is defined by the WHO as an approach to care that consciously adopts individuals’, carers’, families’ and communities’ perspectives as participants in, and beneficiaries of, trusted health systems that respond to their needs and preferences in humane and holistic ways” [6]. There is plenty of evidence for the opportunities of this service model [6–11] as illustrated in the three following examples. In the first one, the Majigi polio campaign in Nigeria, a strategy centred on understanding peoples’ beliefs and engaging traditional, religious and political leaders at all levels led to a relative increase of 310% in the uptake of polio vaccination [12, 13]. In the second one, the Brazilian Family Health Strategy, a model guided by the principles of PCC, resulted in better ratings for care continuity with a more appropriate utilization pattern of care services than the traditional model [14]. Lastly, the PCC approach to improve maternal and neonatal health in El Salvador enhanced community capacity, ownership, and leadership, improved relations with health services, strengthened intersectoral links and coordination mechanisms and resulted in a drop in maternal deaths to zero since 2006 in 90% of the municipalities involved [15].

However, not all people-centred health care initiatives were sustainable or successful. A project in Tanzania that depended on the motivation of a single person in each community did not have a long-lasting impact [16]. In India, an intervention limited to structural health system changes neglecting the health care processes failed to advance the project’s objective of reducing maternal mortality. Its strategy to achieve zero preventable maternal deaths by 2030 was based on structural adjustments related to infrastructure and health workforce without working on positive interpersonal behaviour, information sharing and responsiveness of care [17, 18].

People-centred health care initiatives can start at each level, but often need a demonstration project to document their feasibility and impact, to build consensus, and to convince decision-makers of the utility of broader change [15, 19]. The presented cases show that the involvement of multiple stakeholders is an essential part of PCC. A recent paper affirms the need for this multi-stakeholder collaboration where political and clinical leadership is aligned. Politicians are virtually powerless to effect meaningful change in health care until the health workforce fixes the way care is delivered [8, 20]. A health workforce that is involved at all stages of policy and health service change guarantees accountability, transparency and ownership [9, 21]. Involving all stakeholders in the design, implementation and monitoring of PCC services is a prerequisite. There are many ways to involve stakeholders, from personalised letters used in Serbia to large-scale conferences and meetings bringing together a network of professionals in Sweden and Lithuania [19].

In this paper we present a participatory consultation method to fulfil this purpose. The concept mapping process used in this research stimulates egalitarian participation, understanding and dialogue [22]. Participants’ input is generated and structured in a way that enables data analysis and prioritisation. The generated data can be used for program design and to develop measurement tools to monitor progress towards PCC. This concept mapping methodology has been used both in low, middle and high income countries for conceptualisation, needs assessment, evaluation and program design [23–28]. This method enables a broader perspective on the people-centred health care concept than earlier frameworks [11, 29] by consulting health professionals and laypersons from culturally and socioeconomically diverse backgrounds, as well as students and graduates from public health masters programs. This method goes beyond a theoretical analysis, capturing the tacit knowledge of these diverse stakeholders.

Given the international interest and potential power of people-centred health care, but also the need to understand from all viewpoints how people and not health services can retake the central position towards their health, this study explored laypersons’ and health professionals’ perspectives on policy options and interventions to achieve people-centred health care services.

## Methods

The strategy of this study was to obtain the most comprehensive possible set of perspectives with maximum equality of input on what is needed to achieve people-centred health care services.

### Participants

Participants were recruited over the course of 2012 in Ghent and Antwerp (Belgium) but with a special effort to include visiting staff and temporary residents from a broad range of countries. Purposive sampling was used in order to reach maximal variation regarding people’s perspectives on PCC services. All participants had to be over 18 and comfortable in expressing themselves in Dutch, French, English or Spanish. Diversity was aimed for regarding age, socio-economic status, ethnicity and health status (only for laypersons). From the 60 invited participants, 20 laypersons and 40 health professionals, 53 participated in the study: 16 laypersons and 37 health professionals. They were organised in seven concept mapping workshops: four with health professionals and three with laypersons.

#### Recruitment procedure

To maximize participants’ diversity and to obtain a more universal perspective, different recruitment strategies were used. Laypersons were recruited at social services, at well-baby clinics (free early childhood preventive care) and at the University Hospital of Ghent. An information leaflet was used to invite and inform them. From the 20 laypersons selected who agreed to participate, four people did not show up, despite a telephonic reminder two days before the workshop took place. Participants completed a brief survey on their demographics, schooling and linguistic/ethnic origin to facilitate subsequent diversification of participants. Health professionals were recruited at the Institute of Tropical Medicine Antwerp (public health), at the Department of Family Medicine and Primary Health Care at Ghent University and through disease prevention and health promotion services in Ghent. These health professionals included visiting staff and temporary residents from a broad range of countries. All participants in this study signed an informed consent form.

### Data collection

#### Design

**Fig. 1:**
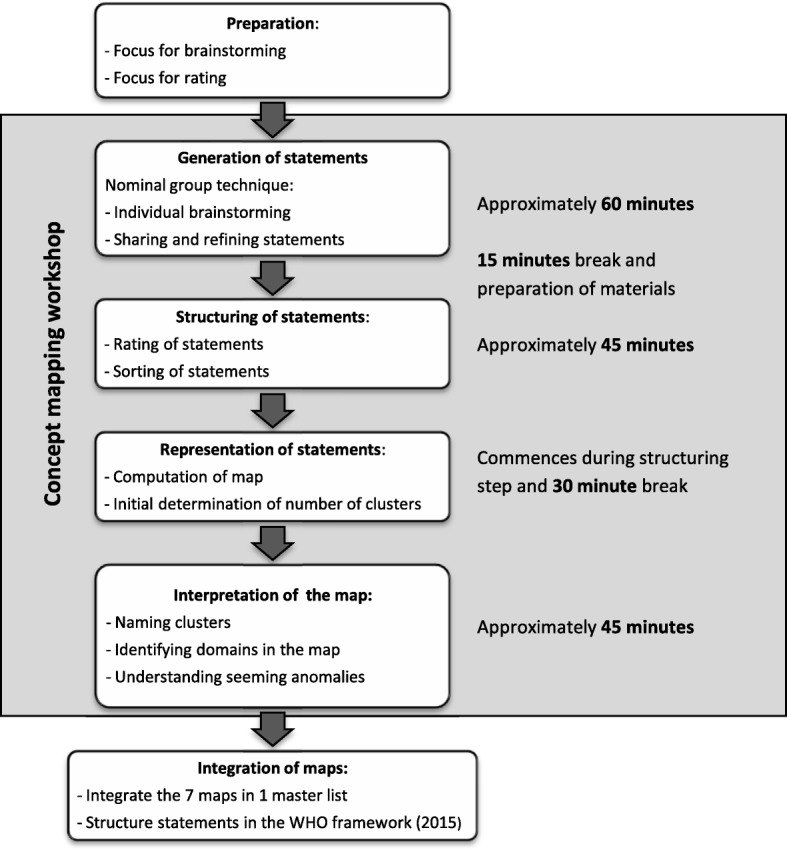
The concept mapping process (adapted from Trochim W, 1989)

The concept mapping methodology by Trochim was selected for its ability to bring together diverse groups of stakeholders and to expeditiously create an interpretable conceptual framework that can serve as a foundation for planning and/ or evaluation. Concept mapping is a highly structured qualitative group method for brainstorming and idea sharing [22]. It consists of a facilitated multi-step process (see Fig. 1) that starts with an open question forming the focus for brainstorming. Statements are generated by the nominal group technique. In this technique each participant individually creates a list of statements and shares them one by one in subsequent rounds with the group. Discussion about the content, relevance or priority is not allowed at this stage [30]. In the next step each participant rates individually the importance of each statement as well as the relations between them. The collective result of this individual structuring is visualised in a concept map. The interpretation and refinement of this concept map with the same group adds to the ownership of the final result.

##### Developing the focus for brainstorming and rating (preparation)

The preparation phase for the concept mapping workshops is fundamental to obtain relevant output. During this phase the “seeding question”, or focus for brainstorming, was developed with an ample and unambiguous focus, guaranteeing its understanding by people unacquainted with the people-centred health care concept and at the same time avoiding a replication of known theories by people knowing the concept. This seeding question was validated linguistically and culturally with a concept mapping expert, two laypersons and two Dutch, two French and two Spanish speaking health professionals. For health professionals the question was: ‘Thinking as broadly as possible, what is needed to enable health care provision, including health promotion and disease prevention, to meet the health and life needs of all people in their life circumstances?’ and for laypersons: ‘Thinking as broadly as possible, what is needed in order to have health care services and orientation to improve your health or to avoid that you get sick that is adequate for you, your family and people you know?’ The focus for rating was: ‘How important or unimportant is it for each of the following statements to be in place to ensure healthcare provision meets the needs of all people in the community?’, with five answer categories ranging from 1 = Not important at all, to 5 = Essential (success is very unlikely without this). The questions for health professionals were available in four languages (Dutch, French, English and Spanish).

##### Generation of statements

The concept mapping workshops started with a brief explanation of the study by the trained facilitators and the signing of the informed consent forms. Subsequently the seeding question was shortly discussed giving the participants the possibility to reword the question and exemplify where needed to reach full understanding. Participants were encouraged to think beyond the Belgian context while annotating individually as many statements as they could think of in response to the seeding question (10 min). Subsequently, they shared their statements within a nominal group process [30] guaranteeing maximum equality of input. The facilitator used paraphrasing, where necessary, to avoid ambiguity or to summarize the idea into a single statement. Descriptive wordings were selected over broad terms like “empowerment”, making it possible for non experts to understand the expressed ideas. A second facilitator annotated all statements, where possible in the participant’s words. The expected advantage of the face-to-face meeting was that the answer of one participant could inspire other participants to generate additional statements. To avoid apprehension of participants in expressing their ideas, laypersons and health professionals participated in separate concept mapping workshops. The statement generation for one health professional workshop was web-based supported by survey monkey, an online survey development software. Ambiguities in the online generated statements were clarified by e-mail.

After this brainstorming session, that took on average 60 min, a 15 min break was needed to prepare the materials for the second part of the workshop.

##### Structuring of statements

In this part each participant received sheets that contained the statements their group had just generated with the task to rate the importance of each statement in response to the focus for rating. They received the same statements printed on individual cards and were asked to sort all statements into groups, clustering statements in a way that made sense to him or her. The minimum number of groups allowed was two and each group was given a name, for example “community participation”. These individual tasks took approximately 45 min after which a second 30 min break was needed.

#### Analysis

##### Representation of statements

During this break the rating and sorting data were inserted in the dosbox version of the concept mapping software of Trochim provided by a concept mapping expert; a commercial version [31] and an open source version [32] are also available. This software allows for a graphical representation of the statements as a concept map. To draw the map, non-metric multidimensional scaling was used to organise the statements in a two dimensional image wherein statements with a higher iterative correlation, sorted together more often, were closer together [33]. The clusters were subsequently formed using Ward’s hierarchical cluster analysis [34] on the X-Y coordinate data obtained from the multidimensional scaling. The desirable number of clusters depended on the specific map, but as a rule of thumb was calculated as number of statements generated divided by five.

##### Interpretation of the map

**Fig. 2:**
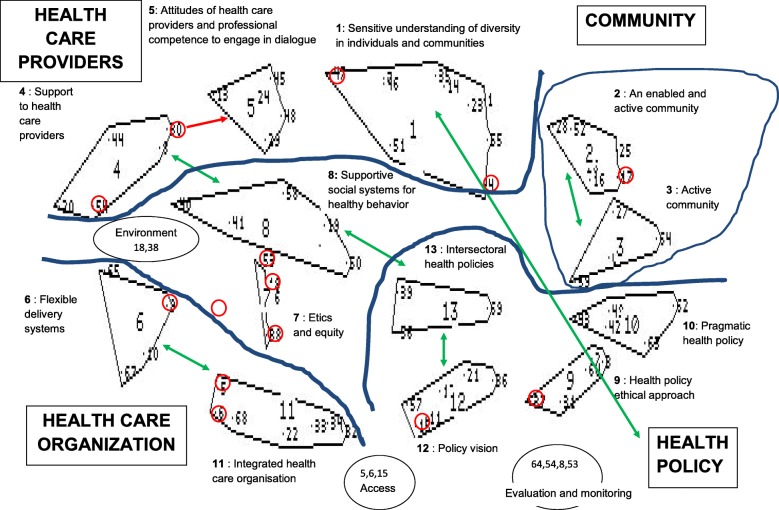
Interpreted concept map of workshop 1: 69 statements, 13 clusters and four domains. Small numbers represent statements; larger numbers are assigned to the clusters; the bold letters indicate the domain names. Statements in a red circle are seeming anomalies that belong to a different cluster. The red arrow indicates a more appropriate cluster for seeming anomalies. The green double arrow indicates relations between different clusters

The statements were sorted in the computed clusters and printed for each participant. Each participant individually was asked to suggest names for the label of each cluster and to revise if some statements should be moved to a more appropriate cluster. Subsequently the map was interpreted by the group: clusters were labelled, erroneous clustered statements were moved, relations between clusters were identified and clusters were grouped into meaningful “domains”. Figure 2 shows the interpreted concept map for the first workshop with health professionals (workshop 1) as an example. This last phase took on average 45 min.

##### Integration of maps: The master list and the WHO’ PCC framework

The concept maps produced by each workshop and their statements were joined together in one “master list” or conceptual framework. The position of each statement in this list was oriented by the “clusters” and “domains” assigned in each workshop: similar clusters were joined, double barrelled clusters were split, new clusters were formed and more specific names were assigned. The researchers decided on the final position of each statement through a consensus procedure wherein two researchers separately sorted the statements that did not fit in their assigned cluster and they discussed together with a third researcher the statements sorted in distinct clusters. The organisation of these statements was a prerequisite for their proper analysis.

To compare our results with the existing WHO framework on Integrated people-centred health care services [11], the same statements were structured by the researchers under the five strategies and strategic approaches of the WHO framework. None of the participants were familiar with this framework since it was developed posterior to this study.

## Results

### Socio-demographic data

The seven concept mapping workshops were organised in Belgium and engaged a total of 53 individuals: 16 (10 female and six male) laypersons and 37 (20 female and 17 male) health professionals, 42% were between 20 and 39 years old, 47% between 40 and 59 and 11% were 60 years or older. As their mother tongue they reported: Dutch (36), Spanish (6), Kinyarwanda (4), French (3), English (2), Italian (1) and Peul (1). Among laypersons, five completed a post-secondary degree and 11 completed secondary school. Regarding professionals, one sociologist and 36 health professionals participated, of which 17 were physicians, being 10 family physicians, 10 masters or master students in public health, six preventive or social workers, one epidemiologist, one physiotherapist and one psychologist. Characteristics of the study participants are described in Table 1.

**Table 1:** Characteristics of study participants

Workshop Number	Profile	Number of participants	Visiting or temporary residents	Country of birth	Mean age (years)
1	Health care & social services	7	3	Belgium, The Netherlands, Scotland	45
2	Primary health care or reproductive health	10	5	Belgium, Ecuador, Rwanda	43
3	Public Health	14	8	Belgium, Senegal, Uganda, Cameroun, Nicaragua, Australia	48
4	Health promotion and preventive services	6	0	Flanders (Northern part of Belgium)	41
5	Laypersons middle-class	5	0	Belgium, Rwanda	26
6	Laypersons low-class	5	0	Belgium	52
7	Parents of young children	6	0	Belgium, Rwanda	32

## Domains and clusters identified by multiple stakeholders to achieve people-centred health care (PCC)

The 452 different statements generated in this study represent policy options and interventions to achieve people-centred health care services. These statements were arranged based on the input from the concept maps into 35 conceptual clusters, grouped in four domains: (1) governance & policy; (2) health workforce; (3) integrated health services and (4) patient, person and community empowerment and participation (see Table 2). Laypersons generated statements related to 24 of the 35 clusters, contributing only to half of the clusters belonging to the domain *governance & policy*. For the description of the results we will mainly describe the 11 clusters that contain 15 or more generated statements.

**Table 2:** Domains and clusters identified by multiple stakeholders to achieve people-centred health care (PCC) services

	Health professionals^a^	Lay-persons^a^
1. GOVERNANCE & POLICY (130)		
1. Decent sustainable health policies (9)	1,2,3,4	
2. Health policies adapted to the local circumstances (10)	1,2,3	
3. Intersectoral health policies (24)	1,2,3,4	
4. A common understanding of health across all stakeholders (8)	1,2,3	
5. All stakeholders participate in health policies (10)	1,3,4	5
6. Sufficient earmarked public health funding (4)	1,2,4	
7. Avoid commercialization of health care (11)	2,3,4	5,6,7
8. Affordable health care for all (31)	1,2,3,4	5,6,7
9. A solidarity based broad-gauged health insurance (10)	3,4	6,7
10. Health services for vulnerable groups (13)	2,4	5,6,7
2. HEALTH WORKFORCE (82)		
11. Accountable medical education (6)	2,3	
12. Excellent communication skills and appreciation of health literacy challenges (24)	1,2,3,4	5,6,7
13. Committed and person friendly health providers (15)	1,2,3	6
14. Respectful attitude based on cultural self-awareness (15)	1,2,3	7
15. Comprehension on peoples’ life circumstances and their use of health services (14)	1,2,3,4	
16. High quality health services: scientific based, respecting patients goals (8)	2,3	7
3. INTEGRATED HEALTH SERVICES (112)		
17. Access to the appropriate level of care (13)	1,4	5,6,7
18. Availability of translators in the health service (4)	3,4	7
19. Effective primary health care as the central hub for care (10)	1,3,4	5,7
20. Care continuity over time (6)	3,4	7
21. Interdisciplinary and multidisciplinary collaboration (10)	1,2,3,4	7
22. Integrated health care across prevention, health promotion, cure and care (23)	1,2,3,4	5,6,7
23. Community based health education, health promotion and disease prevention (18)	1,2,3,4	5,6,7
24. Health facilities/ services close to the people (8)	1,2,3,4	
25. Sufficient supplies and good equipment (6)	3	
26. Data sharing and digitalization (14)	3,4	5,6,7
4. PATIENT, PERSON AND COMMUNITY EMPOWERMENT AND PARTICIPATION (128)		
27. People know, exercise and respect human and patient rights (5)	1,3	
28. People are enabled to navigate the health system (14)	1,2,3,4	5,6
29. Informal care givers and community representatives participate in health care (20)	1,2,3,4	6,7
30. People are aware of the importance of their active participation in health care (13)	1,2,3,4	5,6,7
31. Regular structured interaction between health professionals and the community (10)	1,2,3	
32. Patient’s autonomy is respected and promoted (11)	1,2	5,6,7
33. Adequate and safe nutrition, physical activity and the prevention of diseases are promoted (22)	1,3,4	5,6,7
34. Reliable information is available and unhelpful commercial messages are controlled (16)	1,2,3	5,6,7
35. Health education is attractive and adapted to the characteristics of the target population (17)	1,2,4	5,6,7

Participants identified four domains containing 35 clusters that group 452 policy options or interventions to achieve PCC

The numbers between brackets refer to the number of statements in each cluster

^a^Number assigned to the concept mapping workshops, as described in Table 1

### Governance & policy

For the domain governance & policy, two clusters contain over 15 statements. Firstly, the cluster on *affordable health care for all* that holds the highest number of statements, including 75% rated as very important to essential. This cluster also includes specific strategies that facilitate financial access to health care for vulnerable groups. Some of these specific strategies originate from Belgian experiences like support through social services, health care free at the point of care through community health centres with a capitation system and a minimal contribution through a third party reimbursement scheme. Notwithstanding, laypersons stated that costs still limit access to health care in Belgium. Secondly, the cluster on *intersectoral health policies* that contains only ideas generated by health professionals. This cluster comprises the social determinants of health such as the need to satisfy basic needs like drinking water, safety, a conducive environment with access to parks, transport, etc. and basic education for the whole population. In addition, it includes the needed collaboration between sectors. Other statements rated as essential in the this domain are: the need for long-term interventions, the need for health professionals, planners and policy makers who really listen instead of immediately imposing their own agenda and the need for solidarity to promote the availability of appropriate care for all.

### Health workforce

For the health workforce domain the three most prominent clusters are the need for *excellent communication skills and appreciation of health literacy challenges*, the need to develop a *respectful attitude based on cultural self-awareness* and the need for *committed and person friendly health providers*. Related to communication, both health professionals and laypersons mentioned the need to know the expectations of the patient, to communicate using understandable wording and phrasing and to make time for good communication. Laypersons noted that they often leave the consultation without knowing what will happen next. Related to attitude, health professionals rated as essential to be conscious of their own values and culture and to respect and know patients’ and societal values. To move from attitude to action, the need for committed health professionals with a genuine interest in the patient as a person was mentioned. Factors identified that facilitate this commitment were constructive supervision, self-reflection on the barriers that prevent patient centred care, decent working and living conditions and more equity at the level of the health care provider, such as a reduction in the wage gap between different clinical disciplines. Participants also identified the need to train the health workforce in contextual thinking and inter-professional collaboration and communication.

### Integrated health services

Related to the integrated health services domain the two largest clusters are both linked with prevention and health promotion. The first cluster on *integrated health care across prevention, health promotion, cure and care* refers to the need for integrated health care and not fragmented nor only curative health care. Overall strategies of integration proposed were the creation of bridges between the different services, more investment in and incentives for prevention within health care services and insight in patients’ personal risk profile, personal and family history and life circumstances. Laypersons expressed a need for more nutritional orientation to avoid vitamin or medication use and the need to strengthen aftercare for patients and relatives following cancer treatment or for relatives after events like suicide.

The second prominent cluster is *community based health education, health promotion and disease prevention*. The opportunities of school health, promotion of health through the workplace and the availability of services that support healthy choices like food served at schools were mentioned by both health professionals and laypersons. Health professionals rated as essential to support that healthy behaviour becomes the social norm. Other statements rated as essential in this domain were: good referral and collaboration to guarantee health care continuity, the collaboration between different disciplines and integration between health and welfare.

### Patient, person and community empowerment and participation

In this domain, four clusters contain more than fifteen statements. The first cluster is on the *participation of informal care givers and community representatives in health care*. These community members can assume diverse roles varying from caring for individual patients, peer group support and education, role modelling and enlarging the social capital of a community through links with health and social services. The second cluster is on *promoting adequate and safe nutrition, physical activity and the prevention of diseases*. Health professionals stated the need to enable people to take on a healthy behaviour and lifestyle and to promote a healthy living environment. Laypersons identified the need for prevention, the importance of the Belgian screening program for breast cancer directed to women over 50 years old, but also their felt need to screen in younger age groups, and their need for more information on healthy food choices, vitamins and vaccines. The third cluster is on the *availability of reliable information and the control of unhelpful commercial messages*. Health professionals identified the need for a common understanding regarding where to find reliable information, uniformity in the quality of trustworthy information and a greater control of unhelpful media messages from those with vested financial interests. Likewise, laypersons identified the lack of veracious information on the internet, the need to control commercials related to unhealthy foods and the scarcity of independent health promotion messages versus sponsored commercials on slimming products and vitamins. The last large cluster is on the need for *health education that is attractive and adapted to the characteristics of the target population*. Health professionals mentioned the need to structure the content of health messages as to diminish risk factors while enhancing protective factors, to tailor the message to the specific culture and behaviour of groups by including their participation and to guarantee information channels that are available for everybody like neighbourhood centres, community health centres and other meeting places besides the internet. Laypersons mentioned the importance of mass campaigns on sensitive health issues, like a yearly media campaign on mental health problems in Belgium, as a way to tackle stigma around and facilitate communication about these problems. Both laypersons and health professionals acknowledged the need to look for alternative methods to reach the homeless and immigrants. Other statements rated as essential in this domain were respect for human rights, adequate population knowledge on health care and its organization to improve access, emphasis on education and community information on simple measures that can improve health or prevent diseases and a method of interaction and a regular dialogue between health professionals and the community .

## Multiple stakeholders’ perspective to achieve people-centered health care (PCC) services integrated to the World Health Organization (WHO) framework

Table 3 contains the five strategies and 20 strategic approaches from the WHO framework on integrated people-centred health services. To facilitate the comparison between both frameworks, the 452 statements were rearranged under the WHO strategies and strategic approaches. Statements representing policy options and interventions were identified that related to every single approach. Seven strategic approaches were widely endorsed by all workshops. The results described here will be largely limited to those approaches.

**Table 3:** Multiple stakeholders’ perspective to achieve people-centred health care (PCC) services integrated to the WHO framework

	Health professionals^a^	Lay-persons^a^
1. ENGAGING AND EMPOWERING PEOPLE & COMMUNITIES (132)		
1. Engaging and empowering individuals and families (59)	1,2,3,4	5,6,7
2. Engaging and empowering communities (16)	1,2,3,4	
3. Engaging and empowering informal carers (18)	1,2,3,4	6
4. Reaching the underserved & marginalized (39)	1,2,3,4	5,6,7
2. STRENGTHENING GOVERNANCE & ACCOUNTABILITY (35)		
5. Bolstering participatory governance (18)	1,2,3,4	
6. Enhancing mutual accountability (17)	1,2,3,4	6
3. REORIENTING THE MODEL OF CARE (105)		
7. Defining service priorities based on life-course needs, respecting social preferences (21)	1,2,3,4	6,7
8. Revaluing promotion, prevention and public health (45)	1,2,3,4	5,6,7
9. Building strong primary care-based systems (24)	1,2,3,4	5,6,7
10. Shifting towards more outpatient and ambulatory care (6)	4	6,7
11. Innovating and incorporating new technologies (9)	1,3	5,6,7
4. COORDINATING SERVICES WITHIN AND ACROSS SECTORS (59)		
12. Coordinating care for individuals (16)	1,3,4	5,6,7
13. Coordinating health programs and providers (10)	1,2,3,4	7
14. Coordinating across sectors (33)	1,2,3,4	6,7
5. CREATING AN ENABLING ENVIRONMENT (121)		
15. Strengthening leadership and management for change (3)	1,4	
16. Strengthening information systems and knowledge (11)	1,2,3	5,7
17. Striving for quality improvement and safety (11)	1,2,3	6,7
18. Reorienting the health workforce (50)	1,2,3,4	5,6,7
19. Aligning regulatory frameworks (9)	1,2,3,4	5,7
20. Improving funding and reforming payment systems (37)	1,2,3,4	5,6,7

The 452 policy options or interventions to achieve PCC are sorted under the five strategies and 20 strategic approaches from the WHO framework on integrated, people-centred health services (WHO, 2016)

The numbers between brackets refer to the number of statements sorted under each strategic approach

^a^Number assigned to the concept mapping workshops, as described in Table 1

The first strategy, engaging and empowering people & communities, includes two widely endorsed strategic approaches. The first is the first strategic approach on *engaging and empowering individuals and families* and it contains the largest number of statements, originating essentially from the fourth domain on community empowerment and participation. Nevertheless, seven statements originate from the 12th cluster on communication skills and health literacy challenges and five from the 26th cluster on data sharing and digitalisation. The second is the fourth strategic approach on *reaching the underserved and marginalised* and groups statements originating predominantly from cluster three on intersectoral health policies, cluster eight on affordable health care for all, cluster 10 on health services for vulnerable groups and cluster 24 on health services close to the people.

The WHO strategy on Strengthening governance & accountability received input nearly exclusively from health professionals. All statements but two, sorted under *bolstering participatory governance,* originate from the first domain on governance and policy. The two remainder statements come from the 31st cluster on regular structured interaction between health professionals and the community. The strategic approach on *enhancing mutual accountability* groups statements originating from all four domains, but predominantly from cluster 16 on high quality health services.

The third WHO strategy on reorienting the model of care was commented by both laypersons and health professionals. The eighth strategic approach on *revaluing health promotion, disease prevention and public health* contains statements that were originally sorted under the third domain on integrated health services and the fourth domain on community empowerment and participation. The ninth strategic approach on *building strong primary care-based systems*, gathers statements mainly derived from the third domain on integrated health services and more specifically from the 19th cluster on effective primary health care and the 21st cluster on interdisciplinary and multidisciplinary collaboration. However, eight statements belonged to the second domain, health workforce, principally from the 15th cluster on comprehension on people’s life circumstances and their use of health services.

For the fourth WHO strategy on coordinating services within and across sectors, 33 statements were sorted under the 14th approach on *coordinating across sectors.* Of these 33 statements, 17 originate from the third cluster on intersectoral health policies, while the remainder statements stem from seven different clusters with the 23rd cluster on community based health education, health promotion and disease prevention as the most prominent one.

The fifth WHO strategy on Creating an enabling environment includes the strategic approach that gathered the second most number of statements, being the 18th approach, *reorienting the workforce*. More than 75% of the statements sorted under this approach originate from the domain on health workforce with the greatest contribution from the 13th cluster on committed and person friendly health providers. The remainder of the statements come from two clusters belonging to the first domain on governance and policy, being the fourth cluster on a common understanding of health across all stakeholders and the seventh on the avoidance of health care commercialization. Lastly, the strategic approach on *improving funding and reforming payment systems* is comprised of statements originating exclusively from the first domain on governance and policy. The most prominent clusters contributing to this approach are the sixth on sufficient earmarked public health funding, the eighth on affordable health care for all and the ninth on a solidarity based broad-gauged health insurance.

## Discussion

The findings from this study illustrate that although the conceptualization of people-centred health care recently has been championed by the WHO, a broad range of prerequisites to work towards this global strategy can be elicited from other health professionals and laypersons through a relatively basic and time efficient consultation method. Moreover the results from this study illustrate the divergent perspectives between health professionals and laypersons towards what is needed to achieve PCC.

Exploring the first domain governance & policy*,* we note the relative absence of laypersons’ input in clusters related to policy aspects that surmount financial access to health care services. This corresponds with existing literature which states that few communities understand social determinants of health well enough to start advocating for addressing them at the systems level [35]. Consequently, informing the broader public on all the factors that impact the social determinants of health can leverage community engagement at the policy level. This is critical for the success of interventions to work towards PCC identified at this level like a participatory governance method, intersectoral health policies and the possibility to adapt health policies to the local circumstances. As stated in workshop 1 (see Table 1): *A network between research, decision makers, institutions, civil society, private sector and insurance is needed to ensure effective knowledge exchange and to promote balanced (including interests) policies.* Other pivotal preconditions towards PCC identified in this domain were sufficient public health funding, avoiding commercialization of health care and commitment to affordable health care for all. Specific actions suggested in this domain originate from the Belgian experience being the benefits of a solidarity based broad-gauged health insurance and community health centres with a capitation system, universally accessible, though paying specific attention to vulnerable groups. Furthermore, this integrated needs-based capitation system promotes not only disease prevention and health promotion or a shift from patient to person centred care but also community oriented primary health care (COPC). This later includes community diagnosis and advocacy on the social determinants of health, completing the shift towards people-centred health care [29, 36, 37].

Moving to the second domain on health workforce, the preconditions described here call for action to revise the curriculum of universities and institutes [38] forming the health workforce as well as the mechanisms that subsequently provide continuing education, supervision and accreditation in the field. Consistent with the literature, health professionals must be formed as enlightened change agents, who are agile and quick to adapt their work to the ever changing needs of people at the local, national and international level, acting as expert facilitators. [39, 40] In order to do so, they must comprehend health literacy challenges [41], social determinants of health and the relations between the individual, the family and the community [42], among others. This changing educational need corresponds with the trend toward competency-based learning where emphasis is put on a comprehensive professional education including skills, attitudes and knowledge based on values and ethics [43].

The third domain, integrated health services, was defined by the WHO in 2008 as “The organization and management of health services so that people get the care they need, when they need it, in ways that are user friendly, achieve the desired result and provide value for money”. Other meanings included in the same WHO report were integrated policy-making and management and working across sectors [44]. This definition is very similar to the former definition of people-centred care (WHO, 2010) described as care that is focused and organised around the health needs and expectations of people and communities rather than on diseases [15]. So is there a need to make a distinction between people-centred care and integrated health services? According to the results of this study, where no reference was made to the concept of integrated health services, analysing the prerequisites for people-centred care brings about the need for integrated health services, illustrated through the generation of 112 statements. The many actors that constitute integrated health services were recognised in this domain. For example, there are the politicians who define how people can access the health system and how access to medication, health technology and information systems is regulated. At the local level, there are the actors who mobilize resources and establish multi-stakeholder networks to design, implement and monitor a relevant integrated health system. The latter, which includes the integration of community or public health and individual health care, appeals for effective collaboration and coordination [45]. Revising the literature, the integration of primary care and public health in health networks is challenging and a third party can facilitate this process [46]. Systemic-level factors influencing collaboration include: government involvement, policy that fits with local needs, funding and resource factors, power and control issues, and education and training. A common agenda; adequate knowledge and resources; leadership, management and accountability; geographic proximity of partners; and shared protocols, tools and information sharing are all influential at the organizational level. Interpersonal factors include having a shared purpose, philosophy and beliefs, clear roles and positive relationships, and effective communication and decision-making strategies [47, 48]. Experience with these health networks, defined as Primary Care Zones, exists in Estonia and is under development in Belgium [45, 49].

The last domain patient, person and community empowerment and participation contains the word ‘patient’, referring to ‘an individual awaiting or under medical care and treatment’ [50] thereby finding himself in a vulnerable position. This position is protected through patients’ rights, patient education and informed choices in decision making in the individual care process. For persons living with one or more chronic conditions, self-management support, peer group support [51] and personal goal setting [52] are additional assets to enhance life quality and autonomy. This autonomy changes the focus from a patient acted upon in a biomedical model to a whole person who takes control over his health and life, mobilizing his personal and social resources as well as physical capacities [29, 53]. The clinical encounter becomes the encounter of two experts that have equal power, the person with personal expertise and the health workforce with medical expertise [54]. Person centered care incorporates prevention, health promotion and health education including health system navigation. Health education in its broad sense is known in the literature as ‘health literacy’ and defined as the personal and relational factors that affect a person’s ability to acquire, understand and use information about health and health services [41]. To enhance health literacy in a community, a multilevel intervention is needed that brings attention to the accessibility of health materials and tools, including sectors like education, the communication skills of health professionals, and the institutional characteristics that support the active engagement of patients and communities [55]. Community education is crucial in large parts of Latin America, Asia and Africa where health decisions related to a person are often dominated by the person’s family and community. Education limited to the individual patient would be inadequate in this context. Including people and communities and not only individuals to promote health is paramount since the social determinants of health and their distribution are identified and prioritized in the community and since health is a human right for all. Although, for a community, health education is insufficient to empower people to take control over what determines their health. The community needs enabling social, economic and environmental conditions and real power [56]. In this sense ‘empowerment’ requires real democracy. Furthermore it is important to reach out to people that do not enter the formal health system or that live at the margin of society. Their representation in decision making related to their health must be guaranteed [57, 58]. Direct links between community participation and health outcomes were not found in the literature, but the importance of the process to obtain community uptake, ownership, health equity and sustainability were observed [57, 59, 60].

Of course there are bountiful ways to structure the statements and it is noteworthy to mention the dimensions not strongly related to one of the four domains, which appeared as clusters in the centre of the concept maps due to the variety of ways participants sorted them. Two of these central dimensions were mentioned in nearly all workshops. Firstly, equity, classified under each of the four domains and identified as a prerequisite for needs based care. In all workshops the need for special efforts for vulnerable groups was proclaimed, making services more relevant by adapting them to the specific needs of these groups. A Canadian study identified that primary health care services that included marginalised populations should explicitly address social determinants of health, provide respectful, empowering and culturally competent care tailored to the patient’s and population’s context [61]. Within general health services more investment is needed for people with more needs [10]. This focus corresponds with the concept of proportionate universalism described by sir Michael Marmot, wherein the steepness of the social gradient in health can be reduced by universal actions, but with a scale and intensity that is proportionate to the level of disadvantage [62]. The second central dimension is about the adequate use of technology that facilitates processes, measurements, access, communication, collaboration and information provision. Its potential to improve the effectiveness and equity of care was already identified by B. Starfield in 1998 [63]. How information sharing with the patient and the electronic medical record can advance people centred care is still under discussion [64].

The WHO framework used to validate both the concept mapping methodology and the resulting framework was constructed through a different participatory consultation method. The framework was drafted by a consortium of research institutions and reviewed by experts from the donor community, civil society representatives and through a web based public consultation, among others [11]. Its overlap with the framework identified in this study is compelling.

Seven WHO strategic approaches were widely endorsed by all workshops ratifying their ample recognition by different stakeholders. These approaches perceived as essential to achieve PCC are engaging and empowering individuals and families, reorienting the health workforce, revaluing promotion, prevention and public health, improving funding and reforming payment systems, reaching the underserved and marginalized, coordinating across sectors and building strong primary care-based systems. If we evaluate these strategies, all of them are directly related to changing power relations, giving the population a greater control over their health, putting people and not the health system at the centre. Some approaches are given less weight than expected because laypersons do not recognize their importance such as the strategy on governance & accountability or the approach on engaging and empowering communities. The latter can be due to the sorting of 19 statements related to health education under empowering and engaging individuals and families, while these statements likewise relate to community empowerment. Building strong primary care-based systems became less visible because one of its essential characteristics, coordinating care for individuals, was set as a separate approach. Other approaches seem merely supportive for the seven widely endorsed WHO approaches mentioned above like innovating and incorporating new technologies, strengthening leadership and management for change and aligning regulatory frameworks. The way statements were sorted in this framework can clarify some concepts of this research, but can blur others.

The concept mapping methodology used in this research succeeded in grasping the complexity of the people-centred health care concept. The synergy of the generated ideas with the strategies and strategic approaches identified in the WHO framework on integrated people-centred health care services adds to the validity of the WHO-framework. The participants in this study did not solely generated policy options and interventions, they also assisted in the way these ideas were structured. Since they were structured in a way that seemed logical for each participant individually, the structure can give an idea on how people relate different ideas. As an example, the 10th cluster on health services for vulnerable groups (Table 2) was sorted under the governance & policy domain, while in the WHO framework it belongs under engaging and empowering people & communities (Table 3). While vulnerable groups must be reached in the community and by the community, the decision to include or give priority to vulnerable groups is generally supported and promoted through governance and policy. In this study it seems that participants structured statements overtly related to the actors that are perceived to have the greatest potential to initiate the intervention or policy option making this framework surpass conceptualization towards an action oriented focus.

It is important to note that this study was not intended to capture the entire range of interventions and policy options to work towards people-centred health care services, but it does give a broad picture thanks to the diversity of disciplines, countries of origin and positions in the health system. All participants were encouraged to look beyond the Belgian health care system, but since all laypersons lived in Belgium they related mostly to the Belgian context. As such, this framework does not pretend to represent the universal preconditions to achieve PCC services nor preconditions limited to the Belgian context. Additionally, due to the formulation of the brainstorming focus that explicitly mentions health promotion and disease prevention, statements related to this area are well presented.

The generated framework in this research can assist in defining indicators to measure the level of people-centred care in health policy, health services, pre and postgraduate formation of the health workforce and in the community. It can be instrumental in working towards a consistent and practical international PCC action framework balancing complexity and applicability. We suggest to perform similar studies in low, middle and high income countries with stakeholders of, and a focus on, a single health care system, preferably with involvement of health authorities. Authorities’ involvement increases the likelihood that the final product, an operational and broadly understood framework to plan and evaluate interventions towards people centred health care, will be implemented. Combining different frameworks from different countries can assist in the identification of universal indicators towards people-centred health care.

## Conclusion

The results show that in order to achieve PCC, participative action is needed at the level of policy, health services, community and health workforce, including many sectors beyond health. A major challenge is the collaboration and coordination between the different stakeholders at each level and between levels, where power, resources and a common understanding must be shared. Central in this understanding is the insight that the health sector currently is monopolizing health creating excessive use of health care services as a resource to improve health. At the same time, other resources related to persons, communities, policies and other sectors that have a great potential to tackle social determinants of health, behaviour and ecological factors are not taken advantage of. The concept mapping methodology used in this study is one of the methods to bring together different stakeholders and to foment their egalitarian and respectful participation. It can assist policy makers, the health workforce and communities in the development, planning and evaluation of people-centred health care initiatives. Working towards people-centred health care is a moral obligation to guarantee the highest achievable level of population health in a way that nobody is left behind.

## Abbreviations

COPC: Community Oriented Primary health Care
PCC: People-centred health care
PHC: Primary health care
WHO: World Health Organization
